# *QuickStats*: Percentage Distribution of Gestational Age in Weeks for Infants Who Survived to Age 1 Year and Infants Who Died Before Age 1 Year — National Vital Statistics System, United States, 2014

**DOI:** 10.15585/mmwr.mm6614a5

**Published:** 2017-04-14

**Figure:**
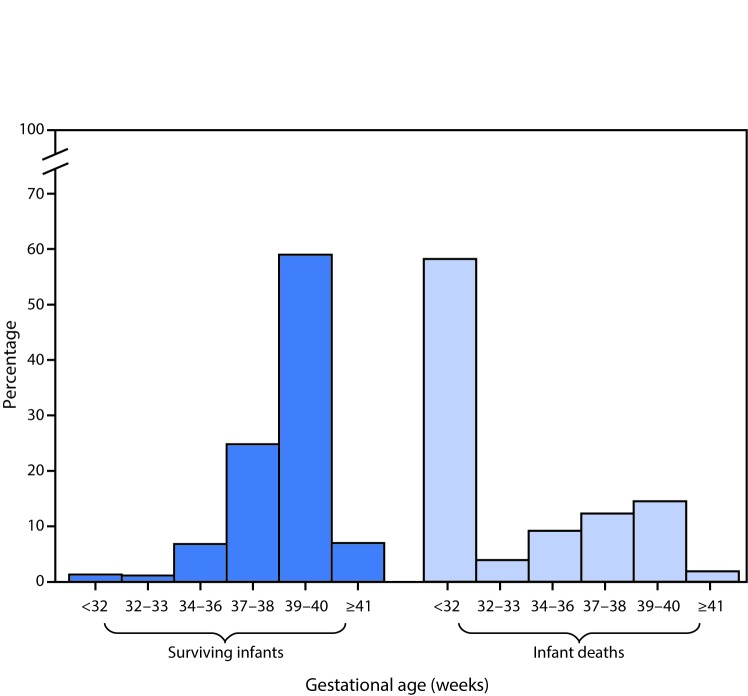
Infants who do not survive the first year of life are more likely to be born at earlier gestational ages. In 2014, 66% of infants who survived to age 1 year were delivered at full term or later (≥39 completed weeks) compared with 16% of infants who died before reaching age 1 year. Fifty-eight percent of infants who died before age 1 year were delivered at <32 weeks gestation compared with only 1% of infants who survived to age 1 year.

**Source:** National Vital Statistics System, Linked Birth and Infant Death Data. https://www.cdc.gov/nchs/nvss/linked-birth.htm.

