# *Erratum:* Vol. 66, No. 14

**DOI:** 10.15585/mmwr.mm6620a8

**Published:** 2017-05-26

On page 392 in “QuickStats: Percentage Distribution of Gestational Age in Weeks for Infants Who Survived to Age 1 Year and Infants Who Died Before Age 1 Year — National Vital Statistics System, United States, 2014,” the second and third sentences of the caption should have read as follows: “In 2014, 66% of infants who survived to age 1 year were delivered at full term or later (≥39 completed weeks) compared with **19%** of infants who died before reaching age 1 year. **Fifty-four** percent of infants who died before age 1 year were delivered at <32 weeks gestation compared with only 1% of infants who survived to age 1 year.”

